# Illigitimacy in France

**Published:** 1913-05

**Authors:** 


ILLIGITIMACY IN FRANCE. 11 per cent. of children born in France are illegitimate—75,000 a year. 20 per cent. are born in Paris, which has about 7 per cent of the population. 600 infanticides and 150 abandonments occur in a year. The mortality of illegitimate children is about double that of legitimate. In plain language, sexual immorality involves manslaughter. Not France alone, but a large transient foreign population must bear this moral responsibility. A law has passed its first reading, making the father of an illegitimate child responsible for its maintenance, but how the parenthood is to be established is a difficult problem. *Medical Press and Circular.*

